# Semisupervised adaptive learning models for IDH1 mutation status prediction

**DOI:** 10.1371/journal.pone.0321404

**Published:** 2025-05-05

**Authors:** Fengning Liang, Yaru Cao, Teng Zhao, Qian Xu, Hong Zhu

**Affiliations:** 1 School of Medical Information and Engineering, Xuzhou Medical University, Xuzhou, Jiangsu, China; 2 Affiliated Hospital of Xuzhou Medical University, Xuzhou, Jiangsu, China; 3 Department of Computer Science and Engineering, State University of New York at Buffalo, Buffalo, New York, United States of America; UC Los Angeles: University of California Los Angeles, UNITED STATES OF AMERICA

## Abstract

The mutation status of isocitrate dehydrogenase1 (IDH1) in glioma is critical information for the diagnosis, treatment, and prognosis. Accurately determining such information from MRI data has emerged as a significant research challenge in recent years. Existing techniques for this problem often suffer from various limitations, such as the data waste and instability issues. To address such issues, we present a semisupervised adaptive deep learning model based on radiomics and rough sets for predicting the mutation status of IDH1 from MRI data. Firstly, our model uses a rough set algorithm to remove the redundant medical image features extracted by radiomics, while adding pseudo-labels for non-labeled data via statistical. T-tests to mitigate the common issue of insufficient datasets in medical imaging analysis. Then, it applies a Sand Cat Swarm Optimization (SCSO) algorithm to optimize the weight of pseudo-label data. Finally, our model adopts U-Net and CRNN to construct UCNet, a semisupervised classification model for classifying IDH1 mutation status. To validate our models, we use a preoperative MRI dataset with 316 glioma patients to evaluate the performance. Our study suggests that the prediction accuracy of glioma IDH1 mutation status reaches 95.63%. Our experimental results suggest that the study can effectively improve the utilization of glioma imaging data and the accuracy of intelligent diagnosis of glioma IDH1 mutation status.

## I. Introduction

Glioma is the most common primary tumor in the central nervous system, accounting for 26.5% of brain tumors, and its 5-year survival rate is 20% to 30% [1,2]. The World Health Organization redefined the classification of gliomas in the 2021 revision of the CNS tumor classification. The revision integrates molecular subtypes for glioma classification, such as IDH-wild type and IDH-mutant [3]. Due to the heterogeneity of tumors, patients with glioma may differ in terms of clinical and pathological characteristics and treatment methods. The integrated molecular subtypes enable doctors to understand tumor types based on molecular expression, providing an important basis for follow-up clinical treatment. The main type of IDH mutation in glioma is IDH1 mutation. IDH1 mutation changes the activity of the IDH enzyme, thereby affecting the treatment and prognosis of patients. Generally, patients with IDH1 mutations have better overall survival and prognosis [4]. Due to the heterogeneity of tumors, patients with glioma may differ in terms of clinical and pathological characteristics and treatment methods. The integrated molecular subtypes enable doctors to understand tumor types based on molecular expression, providing an important basis for follow-up clinical treatment. The main type of IDH mutation in glioma is IDH1 mutation. IDH1 mutation changes the activity of the IDH enzyme, thereby affecting the treatment and prognosis of patients. Generally, patients with IDH1 mutations have better overall survival and prognosis [5,6]. For the above reasons, a noninvasive and easy-to-use method for the preoperative prediction of IDH1 mutation status is essential to alleviate patient suffering and improve the detection efficiency and success rate.

In recent years, we have witnessed tremendous progress in technology development for medical imaging analysis. Currently, deep learning models have been frequently used in almost every phase of medical image processing. Previous research has shown that features extracted from MRI of gliomas are related to gene expression patterns. This has led to a series of studies on mutation status determination. Zhang et al. [7] used the clinical features of multimodal MRI combined with random forest machine learning algorithms to predict IDH mutation status with 86% accuracy. Chang et al. [8] used a convolutional neural network for brain MRI to predict IDH1 mutation status with an accuracy of 94%. Choi et al. [9] used the classification method of segmentation and then combined radiomics features and a convolutional neural network to predict IDH mutation status, obtaining accuracy rates of 93.8%, 87.9% and 78.8% on three different datasets. However, these methods typically involve manual tumor presegmentation and utilize 2D slice classification methods. Adjacent slices of glioma MRI usually share much information. The 2D slice classification method is unable to capture sequential features, and different slices from the same sequence may be distributed in both the training and test sets, potentially introducing bias in the testing phase. In addition, deep learning often requires a large amount of labeled data. However, the abundance of unlabeled data in glioma imaging datasets leads to a significant scarcity of data. The semisupervised learning method, which uses both labeled and unlabeled data to fit the model, improves the accuracy of the model in some cases. However, this method is unstable and may lead to a worse model. In terms of feature reduction, the conventional LASSO model tends to yield sparse results.

**Fig 1:**
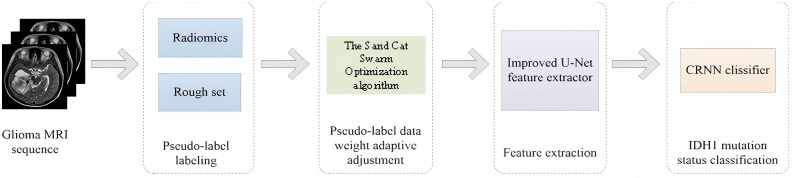
The proposed framework for IDH1 mutation status classification.

To address the above problems, we propose a semi-supervised adaptive deep learning model based on radiomics and rough sets for predicting IDH1 mutation status in glioma MRI images. The specific contributions of this paper are as follows:

(1) To address the problems of data waste and instability in semi-supervised learning methods, we introduce a pseudo-labeling algorithm that removes a large number of redundant radiomics attributes while keeping the pseudo-labeling classification ability unchanged.
(2) A pseudo-labeled data weight adaptive adjustment algorithm using the Sand Cat Swarm Optimization (SCSO) algorithm is proposed to address the potential adverse effects of for-labeled data on the model. The pseudo-labeled data weight adjustment algorithm based on the Sand Cat Swarm Optimization (SCSO) algorithm can adaptively search and capture local and global information, and continuously adjust the search strategy by analyzing the adaptability of each pseudo-labeled data, and ultimately obtain the optimal weights, which effectively reduces the potential adverse effects of pseudo-labeled data on the model.
(3) For glioma image sequences, this paper proposes an improved U-Net-based feature extractor to extract feature sequences from glioma image sequences, and then use a convolutional recurrent neural network (CRNN)-based classifier to classify the feature sequences to predict the IDH1 mutation status of gliomas. The proposed model is an end-to-end model and does not require manual tumor pre-segmentation. In addition, an image sequence rather than a 2D slice classification method is used. The complete framework of the proposed IDH1 mutation status prediction is shown in Fig 1.

## II. Materials and methods

### A. Data collection

The brain MRI data of glioma patients used in this study are from a local affiliated hospital. Informed consent is obtained from all study participants as per their preferences, and the study received approval from the relevant Ethics Committee. Patients came to the hospital for diagnosis from May 30, 2020 to August 31, 2023. Experimental data were acquired on September 1, 2023 and anonymized. All brain MRI scans are preoperative images, including T1C, T2, FLAIR and DWI sequences. Patients who meet the following inclusion criteria are included in this study: (i) histologically confirmed glioma; (ii) pathologic examination of tumor specimens carrie out with proven records of IDH1 mutation status (for patients with known IDH1 mutation status); and (iii) no preoperative treatment. In the end, a total of 316 patients are included in this study, yielding 20,224 images. Among them, 202 patients have a known IDH1 mutation status, with a total of 12,928 images, while 114 patients have unknown IDH1 mutation status, with a total of 7,296 images. Among patients with IDH1 mutation status, 73 are IDH1-mutant and 129 are IDH1-wild type. Among them, level IV account for 55.4%, level III account for 17.8%, level II account for 23.8%, and level I account for 3.0%. Among patients without IDH1 mutation status, 48.9% are grade IV, 23.9% are grade III, 22.7% are grade II, and 4.5% are grade I. To validate the model, we select 80 patients (comprise 45 IDH1-wild type and 35 IDH1-mutant) from the initial 202 patients with known IDH1 mutation status as a validation cohort. In addition, 208 patients with IDH1 mutation status from The Cancer Imaging Archive (TCIA set) are enrolled in accordance with the same criteria. The detail patient characteristics are summarize in Table 1.

**Table 1:** Patient demographics and genetic information.

	Ours (n = 316)		TCIA (n = 208)
	with IDH1 status (n = 202)	without IDH1 status (n = 114)	
Age	50.86 ± 12.15	52.45 ± 12.36	52.05 ± 14.58
Sex			
Male	106	60	110
Female	96	54	98
WHO grade			
I	6	5	0
II	48	26	57
III	36	27	51
IV	112	56	100
IDH1 status			
Wild type	129	/	117
Mutation	73	/	91

### B. Pseudo-labeled algorithm based on radiomics and rough sets

Radiomics [10] involves the high-throughput extraction of extensive information from various images (e.g., CT, MRI, PET). This process transforms images into high-dimensional features and employs data mining techniques to enhance support for medical decision-making. Radiomics can extract thousands of features, many of which are useless or redundant. Therefore, reducing such massive radiomics features is an important preprocessing step for the study. This paper presents a feature reduction method based on radiomics and the positive regions of rough sets.

The experiment used IBEX [11] to extract radiomics features from different sequences of glioma MRI with IDH1 mutation status that are not select for validation, include the gray-level co-occurrence matrix and gray level run length matrix. The gray-level co-occurrence matrix is a matrix function based on pixel distance and angle. It captures a broad spectrum of information within an image, including direction, interval, range of variation, and speed, by calculating the correlation between the gray levels of points at a specific distance and direction in the image. The gray level run length matrix can reflect the comprehensive information of the image gray level, such as direction, adjacent interval and variation amplitude. The parameters for the gray level cooccurrence matrix are configured as follows: GrayLimits=[0 2100], NumLevels = 100, Direction=[0 45 90 135], and Offset=[1 4 7]. The parameters for the gray level run length matrix are set as: GrayLimits=[0 2100], NumLevels = 100, and Direction=[0 90]. The radiomics features obtained based on the gray level cooccurrence matrix and gray level run length matrix are shown in Table 2.

**Table 2:** Image feature information.

Gray level cooccurrence matrix	Gray level run length matrix
AutoCorrelation, ClusterProminence, ClusterShade, ClusterTendendcy, Contrast, Correlation, DifferenceEntropy, Dissimilarity, Energy, Entropy, Homogeneity, Homogeneity2, InformationMeasureCorr1, InformationMeasureCorr2, InverseDiffMomentNorm, InverseDiffNorm, InverseVariance, MaxProbability, SumAverage, SumEntropy, SumVariance, Variance	GrayLevelNonuniformity, HighGrayLevelRunEmpha, LongRunEmphasis, LongRunHighGrayLevelEmpha, LongRunLowGrayLevelEmpha, LowGrayLevelRunEmpha, RunLengthNonuniformity, RunPercentage, ShortRunEmphasis, ShortRunHighGrayLevelEmpha, ShortRunLowGrayLevelEmpha

The radiomics features extracted from MRI data of different sequences constitute the original feature set. First, we perform an independent-sample t test on the extracted features to screen out useless features. When P < 0.05, the parameter is statistically significant. Following the independent-sample T-test, it is possible that the obtained statistically significant features still contain a significant number of redundant features. To eliminate these redundant features, we employ a reduction algorithm based on the positive region of the rough set to reduce the feature set.

The rough set is an attribute reduction method proposed by Pwalak [12] that can quantitatively analyze and process inaccurate, inconsistent, and incomplete information and knowledge. It does not need any prior information other than the dataset needed by the problem. This paper adopts a method based on the positive region of rough set to perform feature reduction in radiomics. There is a large number of features obtained through radiomics methods and hence there is a need to reduce the time complexity and remove redundant features. In comparison to other feature dimension reduction methods, the rough set method ensures that classification accuracy remains the same as the original even after removing redundant attributes. Because the positive region of the rough set is monotonic, the experiment adopted the method of deleting irrelevant attributes one by one. The algorithm begins with the complete set of conditional attributes, and in each iteration, it evaluates an attribute. If the positive region of the decision table remains unaltered after removing the attribute from the set of conditional attributes, the attribute can be excluded from the existing attribute set; otherwise, the attribute is retained. The above operations are repeated until a subset of conditional attributes is obtained. If the positive region of the decision table changes when any attribute is removed, the algorithm ends. The attribute subset at this time is the reduction of the decision table. Finally, the experiment extracts the radiomics features of the unlabeled image data based on the reduced feature set. Then, SPSS is used to calculate Youden’s index for these features, and the maximum value of Youden’s index is used as the threshold. We used Youden’s index to determine the threshold for pseudo-labeling, ensuring that only features with high discriminative power were used for labeling. This reduced the likelihood of assigning incorrect labels to unlabeled data. We judge the IDH1 mutation status of the unlabeled image data according to the threshold of these features. The entire pseudo-labeled algorithm based on radiomics and rough sets is shown in Algorithm 1.

**Table:** 

**Algorithm 1:** Pseudo-labeled based on radiomics and rough sets
**Input:** Labeled dataset A and unlabeled dataset B **Output:** Dataset C with Labeled dataset A and pseudo-labeled data
1. Extract the radiomics features of A to obtain the original feature set A_S_. 2. Perform an independent-sample t test on A_S_ to obtain the feature set A_t_ with *P* < 0.05. 3. Use the reduction algorithm based on the positive region of the rough set to reduce the attributes of A_t_ to obtain the feature set A_y_. 4. for *a* in A_y_ 5. from B extract *a* 6. *b* = *a* 7. B_S_ = B_S_ + {*b*} 8. end for 9. Judge B according to the threshold of B_S_ and output the pseudo-labeled dataset B_1_. 10. C = A + B_1._

### C. SCSO-based adaptive weight adjustment algorithm for pseudo-labeled data

To mitigate the impact of inaccurate pseudo-labeled on the model’s accuracy and stability, this paper introduces an adaptive adjustment algorithm for pseudo-labeled data weights based on the SCSO (Sand Cat Swarm Optimization) method. Specifically, it evaluates the fitness of each pseudo-labeled instance and adaptively modifies its weight during the optimization process. This ensures that pseudo-labels with higher confidence (i.e., those that align well with the labeled data) are assigned greater weight, while less reliable pseudo-labels are down-weighted. As a result, the SCSO algorithm minimizes the influence of noisy or incorrect pseudo-labels, enhancing the model’s stability and accuracy.

The Sand Cat Swarm Optimization algorithm [13] is a heuristic optimization technique inspired by the behavioral traits of sand cats in their natural desert environment. It mimics their strategies for finding food and evading threats. The sand cat’s ears possess the remarkable ability to perceive frequencies below 2 kHz during foraging, couple with an incredible proficiency in excavating prey. Its foraging process comprises two phases: searching and attacking prey. The algorithm effectively obtains the local and global information of the population through fewer parameters and steps, adaptively adjusts the search step length and direction in the iterative process according to the adaptation of each pseudo-label, and finally selects the optimal or better weight scheme as the adjust pseudo-labeled weights. Suppose there are N instances in the glioma image dataset, including Nk labeled instances, Xk={(x1,y1),(x2,y2),…,()}, and (N - Nk) pseudo-labeled instances Xl={,…,}. Then, the loss function of the CRNN-based classifier is expressed as:


$$L_{S}=\sum_{i=1}^{N}l\left(f\left(x_{i}\right),y_{i}\right)$$ (1)

In this experiment, the weight of the labeled data is fixed at 1, and the weight of the pseudo-labeled data is adjusted to change the loss function of the classifier. Since labeled instances and pseudo-labeled instances have different weights, the loss function can be redefined as:


$$L_{S}=\sum_{i=1}^{N_{k}}l\left(f\left(x_{i}\right),y_{i}\right)+\sum_{i=N_{k}+1}^{N}l\left(\alpha f\left(x_{i}\right),y_{i}\right)$$ (2)

where α is the weight applied to the pseudo-labeled instance.

To further determine the value of α, the experiment used the SCSO-based pseudo-labeled data adaptive weight adjustment algorithm to find the optimum solution of α. The algorithm initially initializes parameters including the number of sand cat individuals (N), population positions (Posi) and maximum iteration count (T). The primary parameters governing the transition between the exploration and exploitation stages are denoted as R. It is assumed that the sensitivity range (r) of sand cats extends from 0 to 2 kHz. Each individual sand cat updates its position based on the optimal candidate position (α), its current position (Posc),and its sensitivity range (r). Consequently, sand cats are capable of discovering alternative optimal prey positions after the iterative update. The specific algorithm is shown in Algorithm 2.

**Table:** 

**Algorithm 2:** SCSO-based adaptive adjustment of pseudo-labeled data weight
1. Initialize the quantity of sand cat individuals as N and set the maximum iteration count to T. 2. Initialize the population：Posi(i = 1,2,...,N) 3. While t<=T do 4. Update： r、rG、R 5. Check for any sand cats that have exceed the search space and make appropriate adjustments. 6. Calculate the hunger value (fitness) for each individual sand cat and identify the most satiat sand cat during this iteration. (α) 7. For i = 1 to N do 8. Utilize the roulette wheel selection algorithm to obtain a random angle.(0º≤θ≤360º) 9. If | R|<=1 then $\left\{ \begin{array}{c} P=|\text{rand}(cdotx_{best}^{t}-x_{i}^{t}|\\ x_{i}^{t+1}=x_{best}^{t}-r_{2}\cdot P\cdot \cos(\theta ) \end{array}\right.$ %Update the search positions of the sand cats Else $x_{i}^{t+1}=r_{2}\cdot (x_{best}^{t}+rand(cdotx_{i}^{t})$ %Update the search positions of the sand cats End if End for t = t + 1 End While Return the most satiated cat (optimal solution): α

### D. Feature extraction of glioma image sequences and IDH1 mutation status classification

**Fig 2:**
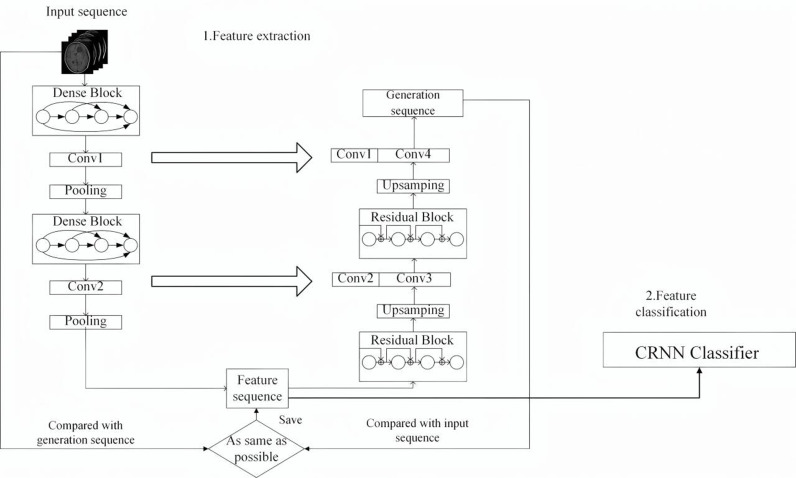
Glioma IDH1 mutation status classification model UCNet.

As shown in Fig 2, we design an IDH1 mutation status classification model UCNet based on an improved U-Net [14] feature extractor and a CRNN [15] classifier using glioma image sequences.

The classification model consists of a feature extractor based on the improved U-Net and a classifier based on a CRNN. The feature extractor based on the improved U-Net is composed of an encoder based on dense blocks and a decoder based on residual blocks, and there is a skip connection between the encoder and the decoder. The use of dense blocks can enhance feature propagation, improve the accuracy and reliability of feature extraction, and reduces the number of parameters. The use of a residual block reduce the weight of some features, improves the effect of MRI spatial sequence reconstruction, and effectively avoids gradient disappearance. Skip connections can effectively merge low-level features and high-level features.

Each dense block is composed of 4 convolutional layers with 64, 64, 128, and 128 convolution kernels. Except for the first and last convolutional layers, the size of the convolution kernel in each convolutional layer is 3*3. The input of each convolutional layer is the sum of the outputs of all previous layers, and each convolutional layer uses LeakyReLU as the activation function. The encoder is composed of two such dense blocks, and the blocks are connected using a 1 * 1 convolutional layer and a 2 * 2 AvgPooling layer to complete downsampling. Each residual block consists of 4 convolutional layers, with 64, 64, 128 and 128 convolution kernels. Except for the first and last convolutional layers, the size of the convolution kernel in each convolutional layer is 3*3. Each convolutional layer uses LeakyReLU as the activation function. The decoder is composed of two such residual blocks and relies on an upsampling layer to increase the size of the feature map; it then stitches them to the corresponding feature map of the encoder.

In this experiment, the glioma image sequence is pass into the encoder to extract the feature sequence, and then it is restore by the decoder. We compare the corresponding pixels of the generated image and the original image. The more similar the final generate image and the original image are, the smaller the loss and the more representative the extract feature sequence. The extract feature sequence is input into the CRNN-based classifier for final classification, and IDH1 mutation status prediction is performe. The CRNN is composed of a convolutional neural network and a recurrent neural network, which is suitable for processing sequence data. In this model, the recurrent neural network part of the CRNN uses long short-term memory. The whole model uses a glioma image sequence, not a 2D slice classification method. Finally, this paper constructs F-UCNet, an IDH1 mutation status classification model based on semisupervised adaptive deep learning. The model uses glioma image sequence data containing pseudo-labeled data, extracts the feature sequence of the image sequence through a feature extractor based on the improved U-Net, and uses a CRNN classifier to predict the IDH1 mutation status. In the classification stage, the model adopts an SCSO-based pseudo-labeled data adaptive weight adjustment algorithm to ensure model stability. The source code is available at the following link: https://github.com/100002006023/Semi-supervised-Adaptive-Prediction-Model.

## Results

### A. Pseudo-labeled based on radiomics and rough sets

This experiment used IBEX to extract 363 radiomics features for each sequence of each labeled patient, of which 330 are derived from the gray level cooccurrence matrix and 33 are derive from the gray level run length matrix. All 363 radiomics features are highly reproducible (ICC > 0.75). Finally, the radiomics features extract from the four sequences of each patient constitute the 1452-dimensional original feature set.

The experiment used SPSS 22 to perform an independent-sample t test on the original feature set. When P < 0.05, a parameter is statistically significant. After the independent-sample t test, there are 372 statistically significant features that constitute a new feature set (T1C 181, T2 56, FLAIR 18, DWI 117). To further remove redundant features from the new feature set, the experiment used a reduction algorithm based on the positive region of the rough set. The software use for attribute reduction is MATLAB 2016.

The features after reduction are shown in Tables 3–6.

**Table 3:** T1C attributes.

Attributes	Youden Index	Threshold	AUC	*P* (<0.05)
135-4Homogeneity2	0.3020	>0.123	0.657	0.039
135-1InformationMeasureCorr1	0.3519	≤-0.341	0.666	0.014
135-4InverseVariance	0.4885	>0.127	0.725	0.005
135-7SumEntropy	0.3993	≤6.257	0.679	0.016

**Table 4:** T2 attributes.

Attributes	Youden Index	Threshold	AUC	*P* (<0.05)
90-1ClusterShade	0.2782	≤-6665.645	0.648	0.048
0-1Homogeneity2	0.4133	>0.6769	0.635	0.035
0-1InformationMeasureCorr1	0.2554	≤-0.385	0.626	0.039
90RunPercentage	0.3788	≤0.422	0.625	0.039

**Table 5:** Flair attributes.

Attributes	Youden Index	Threshold	AUC	*P* (<0.05)
135-4ClusterShade	0.3209	≤-1315.193	0.655	0.022
-333–4Correlation	0.2930	>0.609	0.637	0.044
90RunLengthNonuniformity	0.2910	>2561.888	0.609	0.042

**Table 6:** DWI attributes.

Attributes	Youden Index	Threshold	AUC	*P* (<0.05)
90ShortRunEmphasis	0.3642	≤0.852	0.634	0.047
90-1ClusterShade	0.3953	>-1547.976	0.712	0.004
135-7ClusterShade	0.4804	>-219.698	0.736	0.002
135-7Entropy	0.4116	≤8.155	0.708	0.004
135-7InverseDiffNorm	0.3552	>0.949	0.671	0.030
135-7InverseVariance	0.4534	>0.197	0.716	0.006
90-7SumEntropy	0.4452	≤5.118	0.691	0.006

**Fig 3:**
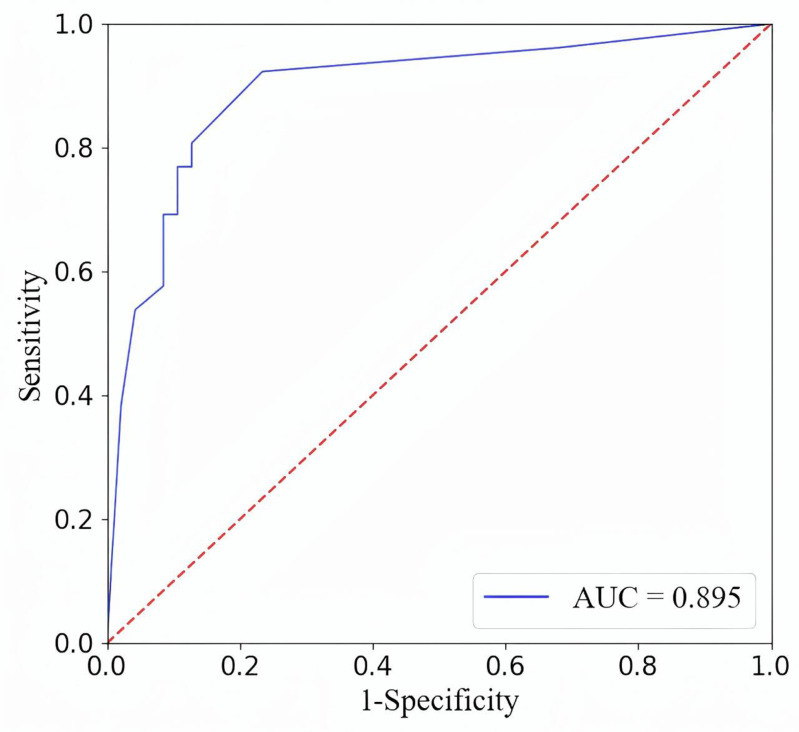
ROC curve of the attribute reduction results.

After attribute reduction, only 18 of the original 1452 features remain. This step greatly reduces feature redundancy, prevents redundant features from interfering with pseudolabeling and improves the efficiency of pseudolabeling. The experiment used the feature set after attribute reduction to classify and verify the labeled image data. The final accuracy rate is 82.19%, the sensitivity is 78.26%, the specificity is 84%, and the AUROC is 0.895 (95% CI). The ROC curve is shown in Fig 3. The accuracy of the conventional semisupervised learning method is 69.05%, and the accuracy of feature reduction by LASSO is 58.33%. Compared with the conventional methods, the method we propose achieved great improvement.

Finally, radiomics feature extraction is performed on 114 pieces of unlabeled image data based on the feature set after attribute reduction. Then, the IDH1 mutation status is predicted with the feature threshold, and the unlabeled image data are labeled. Among 114 cases of unlabeled image data, 75 cases are IDH1-wild type and 39 cases are IDH1-mutant.

### B. SCSO-based adaptive weight adjustment algorithm for pseudo-labeled data

**Fig 4:**
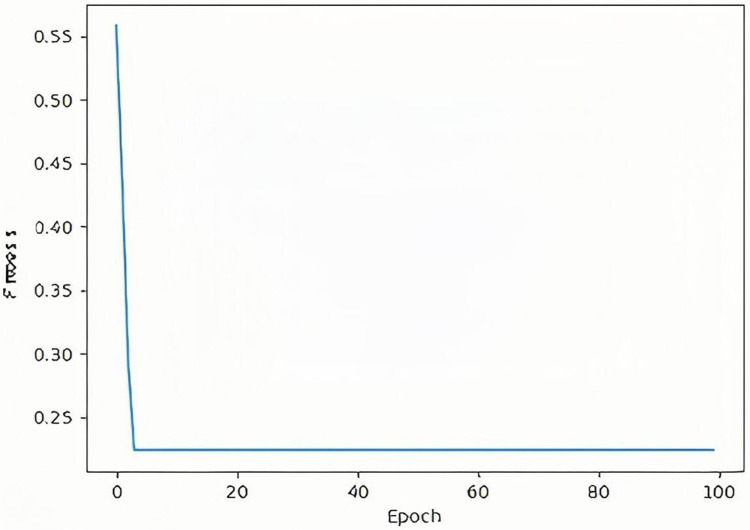
Optimization process of adaptive weight adjustment based on the SCSO.

For the data including the pseudo-labeled data, this experiment adopted the SCSO-based pseudo-labeled data adaptive weight adjustment algorithm. We set Maxgen to 100, and adjusted and output α. The specific optimization process of the classifier of the T2-Flair model is shown in Fig 4. Finally, the optimization ends when the α is 0.2.

### C. Feature extraction of glioma image sequences and IDH1 mutation status classification

We conduct our experiments on a machine with a Windows 10 operating system, a 2.10 GHz Intel Xeon (dual core) processor, 64 GB memory, and 3×GeForce RTX 2080Ti graphics card. We used PyCharm as the development environment, Keras as the deep learning framework, and Python as the programming language.

Image preprocessing included registering images of different sequences to identical 1-mm isovoxel spatial coordinates, performing data augmentation (such as rotation, reflection, flipping, and color adjustment) on the images of some IDH1-mutant cases, and subjecting the images of each sequence to signal intensity normalization. Finally, the images were resampled to sizes of 256 × 256 × 16. These steps ensure consistency across imaging data and enhance the model’s ability to handle variations in spatial resolution, intensity, orientation, contrast, and brightness.

In this experiment, 7 models are constructed for different sequences of glioma images, namely, the T1C model, T2 model, FLAIR model, DWI model, T1C-T2 model, T2-FLAIR model and T1C-T2-FLAIR model. The performance comparisons of these seven models are shown in Table 7.

**Table 7:** Performance comparisons of the seven models.

	Accuracy (%)	Training time (s)
T1C model	93.13 ± 0.72	2072
T2 model	93.75 ± 1.02	2068
Flair model	93.13 ± 1.25	2075
DWI model	91.88 ± 0.72	2132
T1C-T2-Flair model	90.63 ± 0.72	2432
T1C-T2 model	94.06 ± 2.37	2162
T2-Flair model	95.63 ± 0.72	2135

As shown in the above table, when using a single sequence to train the model, the T2 model obtain the highest accuracy rate, follow by the T1C model, and the DWI model had the lowest accuracy rate. Then, two multisequence models, T1C-T2 and T2-Flair, are trained and obtain higher accuracy. When using more sequences, such as in the T1C-T2-Flair model, the accuracy decreases. Therefore, we chose the T2-Flair model with the highest accuracy for subsequent experiments.

**Fig 5:**
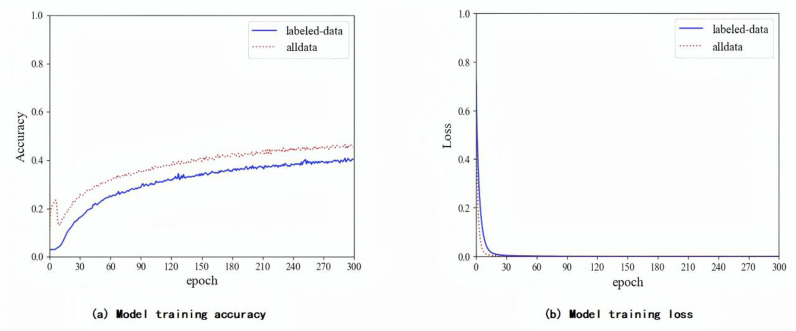
Feature extraction model training process.

First, the feature extractor based on the improved U-Net is used to extract features of the glioma image sequence. In this experiment, the preprocessed glioma image sequence is input into the feature extractor based on the improved U-Net to train the model and extract the required features. Networks are implement use an adaptive moment estimation optimizer and a mean square error loss function. The initial learning rate is set to 10 − 5 with a batch size of 4. Fig 5 shows the process of the T2-Flair model using labeled data to train the feature extraction model and using all data, including pseudo-labeled data, to train the feature extraction model. We stop training the models when the loss curve reaches its lowest point.

As shown in Fig 5, we train the models for 300 epochs. It can be seen from the figures that the accuracy of the feature extraction model trains use data include pseudo-labeled data is significantly higher than that of the model using only labeled data, and the loss convergence speed is much higher. For further comparison, we perform feature extraction on the labeled data and data including pseudo-labeled data and then input the extract feature sequences into the CRNN classifier for training and testing.

**Fig 6:**
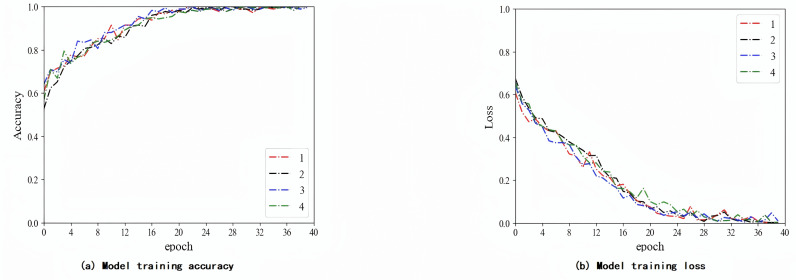
T2-Flair model training process using labeled data.

**Fig 7:**
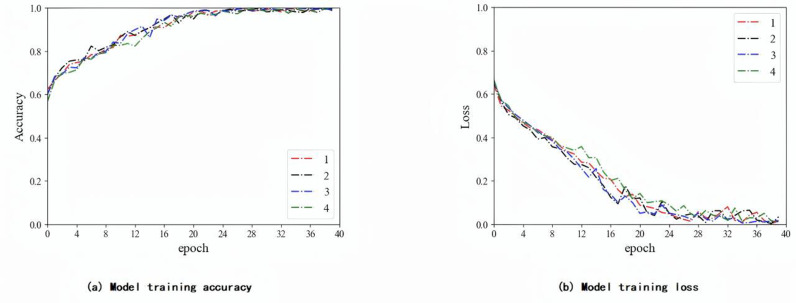
T2-Flair model training process using all data.

**Fig 8:**
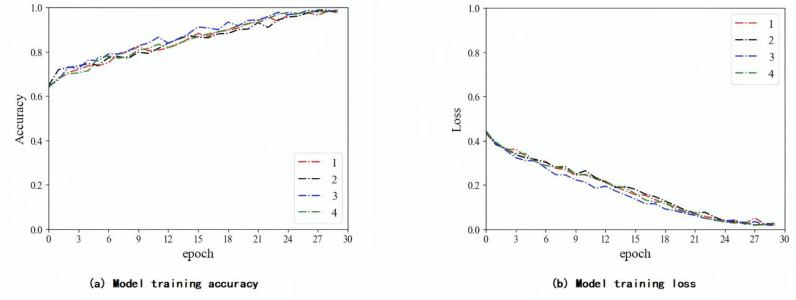
T2-Flair model training process using all data.

In this experiment, the weight of the labeled data is fixed at 1, and the weight of the pseudo-labeled data is adjusted to 0.2 during the classification training process of the T2-Flair model. The CRNN classifier is implemented uses an adaptive moment estimation optimizer and a cross-entropy loss function. The batch size of the UCNet model is set to 40, and the batch size of the F-UCNet model is set to 30. The initial learning rate is set to 10–4. In addition, to avoid overfitting, this experiment used dropout in the CRNN classifier. The dropout ratio is set to 0.5. Then, the model is validated on an independent validation cohort. Figs 6–8 show the training process of the T2-Flair model in three different situations: using labeled data, using all data including pseudo-labeled data, and adjusting the weight of pseudo-labeled data.

As shown in Figs 6-8, the model using the adjusted weights for the pseudo-labeled data converged faster and more consistently. The ablation experiments used data from an independent validation cohort in a local affiliated hospital dataset. Our results are shown in Table 8, where the proposed algorithm achieved 95.63% of the optimal results. As shown in Table 9, we also validated our model using TCIA and compared the results with those published by previous authors. The method in this paper has the highest accuracy of 93.44%.

**Table 8:** The results of the ablation experiment.

Method			Accuracy (%)	NPV (%)	PPV (%)	Recall (%)	F1-score (%)	Training time (s)
T2-Flair-model	pseudo-labeled data	SCSO						
√	√	√	95.63 ± 0.72	93.71 ± 0.02	98.51 ± 0.02	91.43 ± 0.02	94.81 ± 0.01	2135
√	√		91.56 ± 1.20	91.94 ± 0.03	91.44 ± 0.03	89.29 ± 0.04	90.25 ± 0.04	2886
√			90.31 ± 1.20	89.30 ± 0.04	92.64 ± 0.04	85.00 ± 0.07	88.39 ± 0.02	1918

**Table 9:** Comparison with previously published methods for classifying glioma IDH1 status.

	Taha et al. [16]	Choi et al. [9]	He et al. [17]	Choi et al. [18]	Tripathi et al. [19]	F-UCNet (ours)
Accuracy (%)	75.00	78.80	87.60	91.70	91.96	**93.44**

## Discussion

In this study, we propose a semisupervised adaptive deep learning model that uses MRI to predict the IDH1 mutation status of gliomas. Firstly, we propose a pseudo-labeled algorithm based on radiomics and rough sets for a large amount of unlabeled data. The algorithm extracts numerous radiomics features from the labeled data and then employs an independent-sample t test to initially filter out significant features from a vast set of features. To further remove redundant features, the algorithm utilizes an attribute reduction method based on the positive region of the rough set, resulting in a feature set after attribute reduction. The algorithm extracts the radiomics features from the unlabeled data according to the feature set after attribute reduction and labels the unlabeled data according to the threshold of the feature set. Subsequently, to address potential inaccuracies in the pseudo-labeled data, this paper introduces an SCSO-based adaptive weight adjustment algorithm for pseudo-labeled data. Furthermore, we construct an IDH1 mutation status classification model based on glioma image sequences. This model consists of a feature extractor utilizing an improved U-Net and a classifier based on a CRNN. The feature extractor based on the improved U-Net is composed of an encoder based on a dense block and a decoder based on a residual block. The encoder performs feature extraction on the input glioma image sequence, and the decoder restores and outputs the feature sequence. The more similar the generated image is to the original image, the more representative the extracted features become. We input the feature sequence extracted by the feature extractor into the CRNN classifier for IDH1 mutation status classification. In the classification stage, the SCSO algorithm fixes the weight of the labeled data and uses the algorithm to adaptively adjust the weight of the pseudo-labeled data. Then the optimal weight is output. We constructed 7 models for different sequences of glioma imaging, namely, the T1C model, T2 model, FLAIR model, DWI model, T1C-T2 model, T2-FLAIR model and T1C-T2-FLAIR model. The IDH1 mutation status classification models are trained on 122 patients (7,808 images) with IDH1 mutation status and 114 patients (7,296 images) without IDH1 mutation status, and their performance is validated on an independent validation cohort (80 patients).

Prior studies [7–9] utilized 2D slice classification methods based on labeled data, with many of them necessitating tumor presegmentation. However, in reality, there are more data without IDH1 mutation status labels, which causes considerable data waste. Therefore, this paper proposes a pseudo-labeled algorithm based on radiomics and rough sets. Pseudo-labeled and the judicious utilization of unlabeled data can enhance data efficiency and contribute to model accuracy improvement. In addition, the IDH1 mutation status classification model proposed in this paper is end-to-end and does not require tumor presegmentation. The model is divided into two parts: a feature extractor based on the improved U-Net and a classifier based on a CRNN. Incorporating dense blocks and residual blocks in the feature extractor based on the enhanced U-Net effectively mitigates the vanishing gradient issue, enhance the accuracy and dependability of feature extraction. The CRNN-based classifier considers the relationship between the slices and can help the model extract the features between the slices, so it is very suitable for sequence data. Leveraging the SCSO-based adaptive weight adjustment algorithm for pseudo-labeled data significantly expedites model convergence, enhances model accuracy, and ensures model stability. The experimental results show that the performance of the model using all data include pseudo-labeled data is better than that use only labeled data. The performance of the model that adaptively adjusted the weight of the pseudo-labeled data is better than that of the model that did not. In the end, the T2-Flair model obtain the highest accuracy rate for IDH1 mutation status classification, which is higher than that of using only a single sequence. Nevertheless, when additional sequences are included, as in the case of the T1C-T2-Flair model, the accuracy exhibited a decrease.

Our analysis presents an alternative noninvasive method for predicting the IDH1 mutation status of glioma patients. The experimental results demonstrate that the method presented in this paper can enhance the accuracy of intelligent IDH1 mutation status diagnosis and the utilization of glioma imaging data. As a result, this method holds promising applications in alleviating patient suffering, reducing economic burdens, and aid in clinical diagnosis.

Although our algorithm has achieved significant results in predicting IDH1 mutation status, it also exhibits certain limitations. Firstly, the algorithm requires a substantial amount of MRI imaging data for training and validation, which may be constrained by data acquisition and processing in certain scenarios. Secondly, while we conducted model validation in our study, its generalization capability to other datasets or medical centers remains insufficiently validated and is contingent upon the stability of specific equipment and techniques. Additionally, the accuracy of pseudo-labeled data remains a challenge, potentially leading to unstable model training or misleading outcomes. Moving forward, we will further research and refine the algorithm proposed in this paper to enhance its effectiveness and reliability in real-world clinical applications.

## Conclusion

In this paper, we propose a semi-supervised adaptive prediction model for IDH1 mutation status in gliomas. For unlabeled image data, we introduce a pseudo-labeling algorithm based on radiomics and rough sets, and an adaptive weight adjustment algorithm for pseudo-labeled data based on SCSO. For glioma image sequences, we introduce a feature extractor based on improved U-Net and a classifier based on CRNN to predict IDH1 mutation status. Experimental results show that the method achieves 95.63% accuracy in IDH1 mutation state classification. This indicates that the proposed method is reliable and promises to aid in clinical diagnosis. In the future, we will conduct a more in-depth study to address the shortcomings of the algorithm, such as insufficient data size and errors in the accuracy of pseudo-labeled data.
